# Prenatal Diagnosis of Fraser Syndrome at 20 Weeks' Gestation: A Case Report and Review of Literature

**DOI:** 10.7759/cureus.96652

**Published:** 2025-11-12

**Authors:** Shanza Rana

**Affiliations:** 1 Obstetrics and Gynaecology, Mid and South Essex NHS Foundation Trust, Basildon, GBR

**Keywords:** cryptophthalmos, fraser syndrome, genetic counselling, prenatal diagnosis, syndactyly

## Abstract

Fraser syndrome is a rare congenital multisystem disorder, characterised by cryptophthalmos, syndactyly, and urogenital/renal anomalies, with high perinatal mortality. We report a pregnancy in which multiple severe fetal anomalies were identified at the routine anomaly scan (20 + 4 weeks’ gestation), including central nervous system, craniofacial, renal, and limb findings highly suggestive of Fraser syndrome. The mother declined invasive testing and postmortem examination. Following multidisciplinary counselling regarding prognosis and options, the patient opted for medical termination of pregnancy. She experienced an allergic reaction to mifepristone and was managed with a misoprostol-based regimen. A stillborn female infant was delivered; the mother required a transfusion for postpartum haemorrhage due to retained placenta. Suspicion of Fraser syndrome on prenatal ultrasound should prompt multidisciplinary input and offer of genetic counselling. Where molecular or postmortem confirmation is not available, diagnosis may remain presumptive, but anticipatory counselling regarding poor prognosis and recurrence risk is essential.

## Introduction

Fraser syndrome is a rare genetic disorder, which is inherited in an autosomal recessive pattern [1]. It is characterised by prenatal skin blistering, syndactyly (fusion of fingers or toes), and renal abnormalities [2]. Its hallmark feature includes hidden or malformed eyes [3]. Various other anomalies have also been associated with Fraser syndrome, such as maxillofacial, oro-dental, otorhinolaryngeal, cardiac, musculoskeletal, and anorectal malformations [4]. Ocular abnormalities, known as cryptophthalmos, are classified into three forms: complete, incomplete, and abortive [5]. Fraser syndrome is further classified based on ophthalmologic features as isolated cryptophthalmos, cryptophthalmos sequence, and cryptophthalmos syndrome [3]. Unlike Fraser syndrome, isolated cryptophthalmos is inherited in an autosomal dominant pattern [3].

Fraser syndrome can be diagnosed either by prenatal ultrasound, perinatal biopsy, or postnatal clinical examination [6]. The first description of this syndrome was based on clinical grounds, when George R. Fraser in 1962 reported multiple cases presenting with the characteristic triad of cryptophthalmos, syndactyly, and urogenital anomalies [1]. Prenatal diagnosis of Fraser syndrome is made by ultrasound identification of renal agenesis and laryngeal atresia [7]. The first prenatal diagnosis was reported by Feldman in 1985, who identified microphthalmia at 18 weeks of gestation in a fetus with an affected sibling [8]. Postnatal diagnosis is made using the criteria defined by Thomas et al., which require either two major features (cryptophthalmos, syndactyly, genital anomalies, or an affected sibling) with one minor, or one major feature with at least four minor anomalies (including ear, nose, laryngeal, skeletal, cleft lip/palate, umbilical hernia, or renal defects) [9].

Nearly half of infants with Fraser syndrome are either stillborn or die soon after birth. Cognitive delays are common among survivors [10]. Life expectancy is often less than one year [11]. Management of children born with Fraser syndrome should be provided in the specialised medical centres with a multidisciplinary team comprising anaesthetists, ear, nose, and throat specialists/maxillofacial surgeons, and clinical genetics [7].

This case is reported to emphasise the role of routine anomaly ultrasound in the antenatal recognition of Fraser syndrome and the impact of early diagnosis on parental decision-making. Identifying the characteristic constellation of central nervous system, renal, craniofacial, and limb abnormalities at 20 weeks’ gestation allowed timely involvement of a multidisciplinary team. This, in turn, facilitated structured, non-directive counselling about prognosis, management options and recurrence risk, which is essential when caring for families facing a rare, life-limiting fetal condition.

## Case presentation

A 31-year-old woman (gravida 7, para 6; five living children) booked for antenatal care at a district general hospital. Her obstetric history included postpartum haemorrhage after her first and fourth deliveries, an infant death at 15 months due to a brainstem anomaly following her second pregnancy, and an emergency lower-segment caesarean section at 36 weeks in her fourth pregnancy. Subsequent deliveries were uncomplicated. Pre-pregnancy body mass index was 24.8 kg/m², and she had no chronic medical conditions. Her blood group was A Rhesus-negative. She was a non-smoker, reported a penicillin allergy, and had no known family history of genetic disorders. First-trimester combined screening indicated low risk for trisomy 21/13/18 (each ~1 in 5000). Nuchal translucency measured 1.3 mm, pregnancy-associated plasma protein-A was 1.28 MoM, and free beta human chorionic gonadotropin was 0.81 MoM. Routine virology screening was negative.

**Figure 1:**
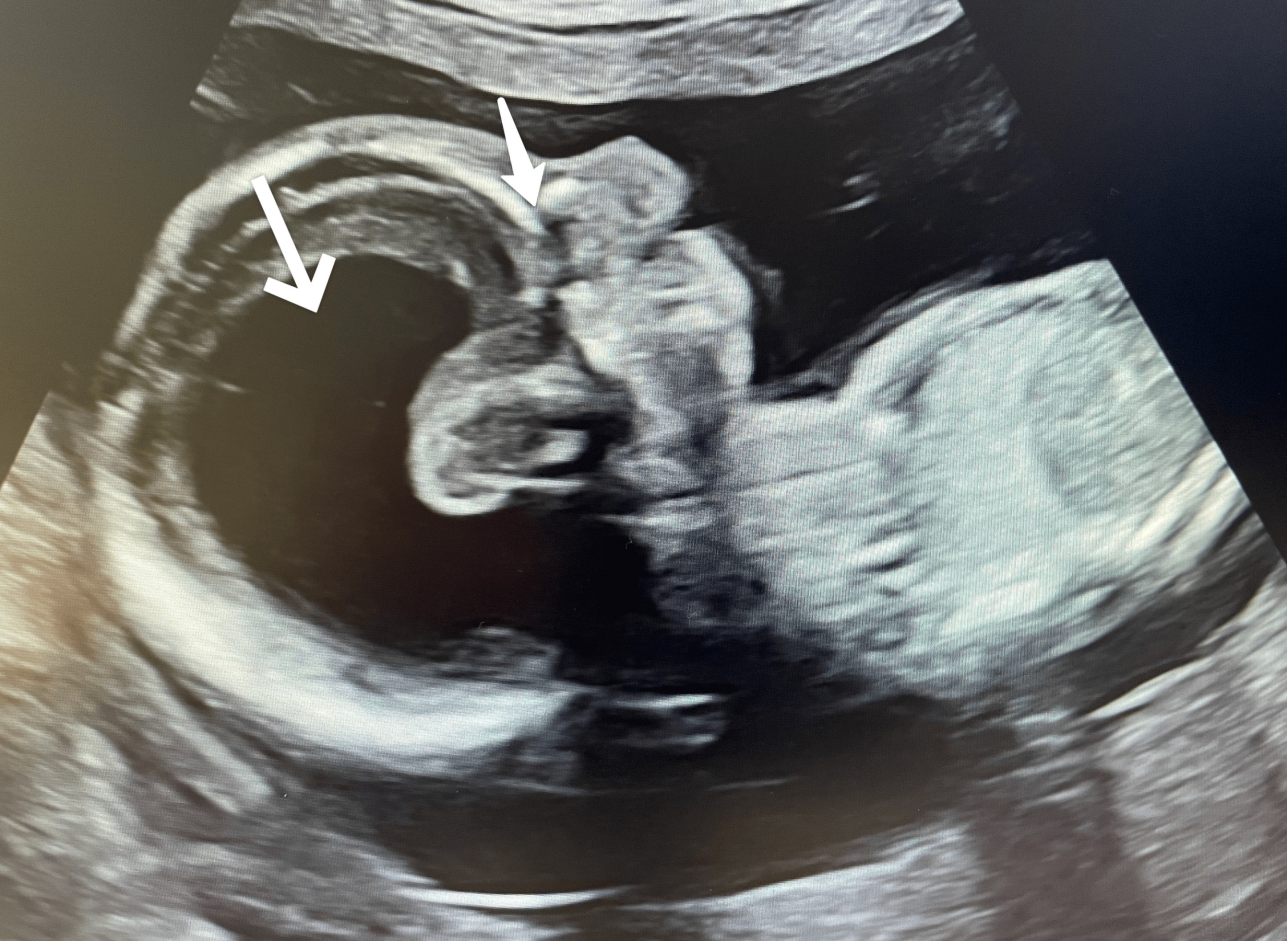
Prenatal ultrasound showing marked ventriculomegaly (lower white arrow) with enlargement of the lateral ventricles and absence of the left eyeball (upper white arrow), consistent with anophthalmia.

At the routine anomaly scan (20 + 4 weeks’ gestation; estimated due date was 30 May 2025 by ultrasound), multiple fetal abnormalities were identified. Prenatal ultrasound demonstrated marked ventriculomegaly and absence of the left eyeball, as shown in Figure 1. Central nervous system abnormalities included lobar holoprosencephaly, severe cerebellar hypoplasia, absence of the corpus callosum and cavum septi pellucidi, and severe ventriculomegaly (>15 mm). Facial findings included cleft lip and palate, left anophthalmia, right microphthalmia with the eye covered by skin, and severe micrognathia.

**Figure 2:**
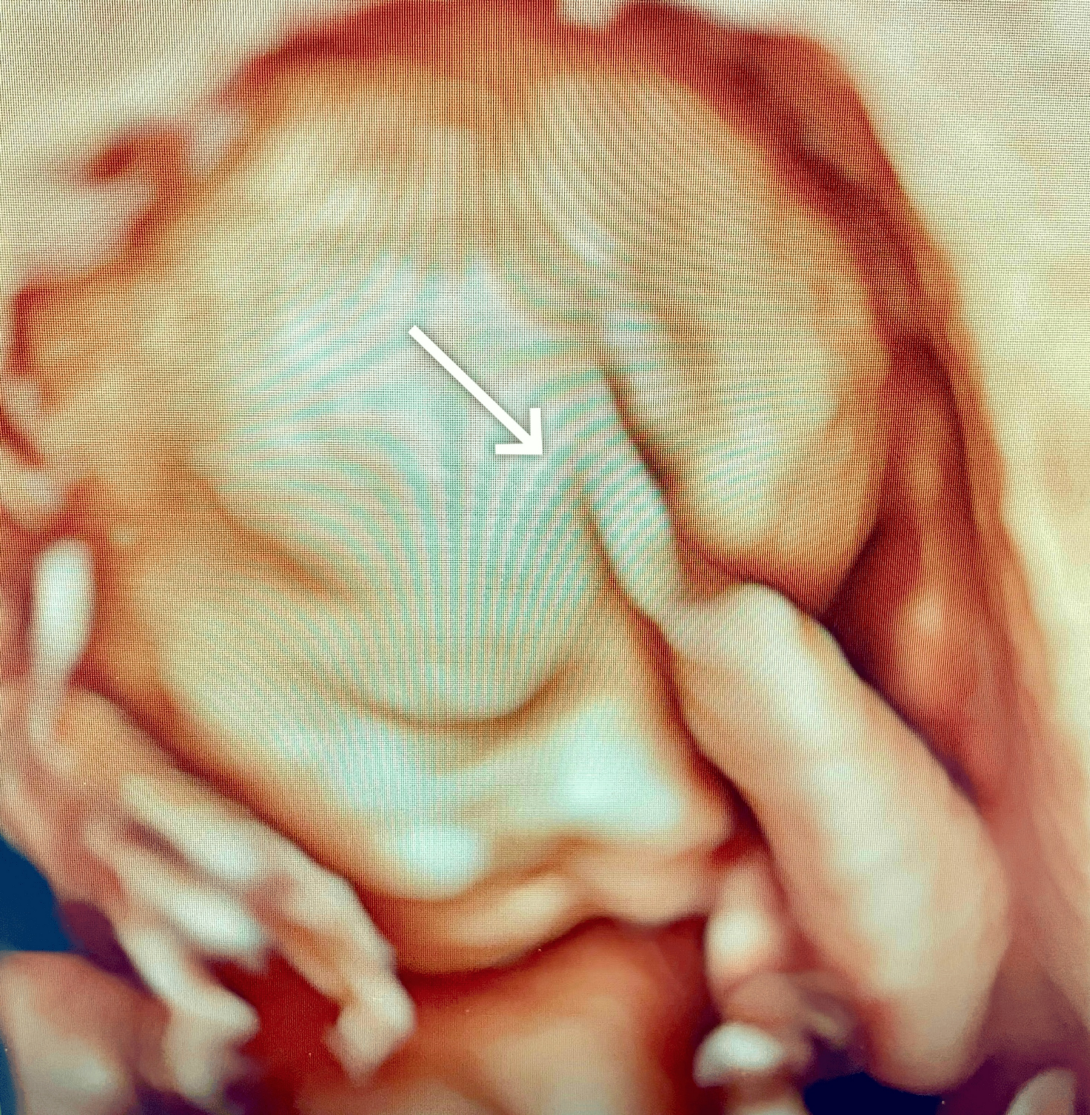
Fetal ultrasound demonstrating craniofacial dysmorphism (white arrow).

The hands showed bilateral syndactyly between the index and little fingers. A 3D fetal ultrasound further demonstrated craniofacial dysmorphism, as illustrated in Figure 2. Cardiac assessment identified a large muscular ventricular septal defect with the left ventricle smaller than the right. The left kidney was not visualised in an empty renal fossa (consistent with renal agenesis). The stomach bubble was absent in the context of polyhydramnios, raising concern for oesophageal atresia. Additional findings included a single umbilical artery, an abnormal craniofacial profile, breech presentation, and an estimated head circumference of >95th centile. This constellation of abnormalities was highly suggestive of Fraser syndrome.

The findings and prognosis were discussed by the fetal medicine team. Options offered included expectant management, associated with a high likelihood of stillbirth or early neonatal death, and termination of pregnancy (TOP). Invasive genetic testing was offered but declined, as results would not alter prognosis.

The patient opted for medical TOP. After an allergic reaction following mifepristone administration, the regimen was changed to protocol-based vaginal misoprostol. After five days of misoprostol, she delivered a stillborn female infant weighing 435 g (head circumference of 21 cm) with a visible cleft lip/palate and dysmorphic facies. Estimated blood loss was 1.5 L secondary to retained placenta, managed with uterotonics and transfusion of two units of packed red cells. The patient declined postmortem examination and postnatal genetic testing.

## Discussion

To date, approximately 250-300 cases of Fraser syndrome have been reported worldwide. This case illustrates a classical presentation of Fraser syndrome detected prenatally. The constellation of CNS malformations, renal agenesis, ocular anomalies, and syndactyly strongly suggested the diagnosis. Literature describes similar findings, with poor prognosis and high perinatal mortality [12].

Once the ultrasound findings raised a strong suspicion of Fraser syndrome in our patient, counselling was undertaken in a dedicated fetal medicine clinic in line with local protocol. The woman and her partner were informed that the abnormalities were most likely due to a serious genetic condition and that the prognosis was extremely poor, with a high likelihood of stillbirth, very preterm delivery, or early neonatal death. Management options included continuation of the pregnancy with expectant care, invasive diagnostic testing to confirm a chromosomal or single-gene abnormality, and medical TOP. The increased risk of miscarriage associated with invasive testing and the fact that a 'normal' genetic result would not alter the overall prognosis were discussed in detail. The couple declined invasive testing, postmortem examination, and further genetic investigations after delivery, and chose medical TOP at their local hospital. This case, therefore, illustrates not only the typical prenatal findings of Fraser syndrome but also the importance of comprehensive, non-directive counselling and shared decision-making.

Our patient declined genetic testing, but given the history of one previous child affected with a brainstem abnormality, autosomal recessive inheritance is highly plausible. Such women should have genetic counselling due to a 25% recurrence risk in future pregnancies [13]. The incidence of Fraser syndrome is estimated at 0.43 per 100,00 live births [14].

Fraser syndrome can be diagnosed during pregnancy, during childbirth or after delivery, but our case demonstrates an antenatal diagnosis due to typical ultrasound features. The management should involve a multidisciplinary team approach; nonetheless, a poor prognosis of the condition limits possible interventions.

## Conclusions

Fraser syndrome is a rare but severe congenital disorder with a poor prognosis. Antenatal diagnosis enables early parental counselling and informed decision-making. This case highlights the role of multidisciplinary management and the importance of offering genetic counselling to affected families.
